# [Retracted] Epithelial cell adhesion molecule is overexpressed in hypopharyngeal carcinoma and suppresses the metastasis and proliferation of the disease when downregulated

**DOI:** 10.3892/ol.2026.15477

**Published:** 2026-02-02

**Authors:** Yakui Mu, Na Sa, Liang Yu, Sumei Lu, Haibo Wang, Wei Xu

Oncol Lett 8: 175–182, 2014; DOI: 10.3892/ol.2014.2140

Following the publication of the above paper, a concerned reader drew to the Editor's attention that, regarding the western blots shown in Fig. 5, the alpha-catenin panel in Fig. 5A (total protein) appeared to be very similar to the beta-actin panel in Fig. 5B (soluble protein). Upon performing an independent analysis of the data in this paper in the Editorial Office, it also came to light that the immunohistochemical staining images featured in Fig. 1A-C contained apparent anomalies, namely the repeated patternings of small areas of cellular data within the same figure panels.

After having conducted this independent investigation of the data in the Editorial Office, the Editor of *Oncology Letters* has determined that this paper should be retracted from the Journal on account of a lack of confidence in both the scientific integrity of the data and the accuracy with which certain of the figures were assembled. The authors were asked for an explanation to account for these concerns, but the Editorial Office did not receive a reply. The Editor regrets any inconvenience that has been caused to the readership of the Journal.

